# Chorea in Hereditary Leukodystrophies – Overview of Two Cases

**DOI:** 10.5334/tohm.1103

**Published:** 2025-12-11

**Authors:** Andona Milovanović, Milica Ječmenica-Lukić, Nina Mazalica, Vanja Radišić, Maja Đorđević-Milošević, Ana Marjanović, Marija Branković, Vladana Marković, Nikola Kresojević, Vladimir Kostić, Nataša Dragašević-Mišković

**Affiliations:** 1 Clinic for Neurology, University Clinical Centre of Serbia, Belgrade, Serbia; 2 School of Medicine, University of Belgrade, Belgrade, Serbia; 3 Mother and Child Health Care Institute of Serbia “Dr Vukan Čupić”, Belgrade, Serbia

**Keywords:** chorea, leukodystrophies, genetics, movement disorders

## Abstract

**Background:**

Leukodystrophies are inherited heterogeneous diseases that are predominantly characterized by degenerative changes in the white matter of the central nervous system. These disorders begin both in childhood and in adulthood and have a complex phenotype that includes involuntary movements specifically chorea.

**Methods:**

This paper describes two female patients for whom generalized chorea was the primary clinical manifestation of leukodystrophy. Literature search was done through PubMed database with aim to included articles that described case reports of patients (both adult and childhood-onset) with leukodystrophy presenting with chorea in patients with metachromatic leukodystrophy (MLD) or L-2-hydroxiglutaric aciduria (L2HGA).

**Results:**

The first case presents MLD with adult-onset chorea combined with cognitive-behavioral changes mimicking Huntington’s disease, while the second case is caused by L2HGA and the diagnosis had been established in the adulthood. The search resulted in 163 articles, but only one in the end described phenotype suggestive of dyskinetic movement disorder.

**Discussion:**

Leukodystrophies, though primarily white matter disorders, can present with involuntary movements. Our cases with MLD and L2HGA highlight adult patients with chorea as a key manifestations, so metabolic and genetic testing is crucial in unexplained chorea.

**Highlights:**

Leukodystrophies cause white matter degeneration and involuntary movements. We present two cases: one with MLD mimicking Huntington’s disease and one with L2HGA diagnosed in the adulthood. These clinical manifestations have not yet been precisely reported in the literature. This manuscript present rare adult-onset chorea in leukodystrophies and expands phenotypic diversity.

## Introduction

Leukodystrophies are hereditary heterogeneous group of disorders predominantly characterized by degenerative changes in the central nervous system’s white matter [1]. These disorders start not only in childhood, but adulthood as well, as complex phenotype, such as dementia, psychiatric signs, spasticity, hyperreflexia, ataxia, seizures, involuntary movements, as well as diverse T2w hyperintensities on the magnetic resonance imaging (MRI) of the white matter in the brain and spinal cord [2]. This variety of clinical manifestations may pose a diagnostic challenge.

Due to recognizing specific patterns of changes on brain MRI, corresponding algorithms of clinical differentiation for these disorders have been made [2].

Involuntary movements are described in leukodystrophies, but in clinical practice, they often remain insufficiently defined [3]. Although they are not usually a presenting sign of the disorder, their recognition is very important since it sometimes directs us in making differential diagnoses, and very often they have a substantial impact on the patient’s life quality.

Metachromatic leukodystrophy (MLD, OMIM250100) represents an autosomal recessive (AR) form of leukodystrophy caused by pathogenic variants in *ARSA* gene. Due to the advancements in next-generation sequencing (NGS), new causing variants in *ARSA* gene are recognized, while the phenotype has been expanded as well, particularly in the cases with adult-onset [4,5].

When occurring in childhood, MLD usually manifests itself in the loss of motor abilities, seizures, ataxia, and optic atrophy, whereas when starting in adulthood, patients exhibit behavioral changes, dementia, and psychosis [5]. Although one of the papers mentions that choreoathetoid movements can seldom be a part of the clinical manifestations, so far there are no detailed case reports and MLD is not considered in differential diagnosis of generalized chorea [6].

L-2-hydroxiglutaric aciduria (L2HGA, OMIM236792) is an AR disorder caused by pathogenic variants in *L2HGDH* gene. Tipically, starts in childhood with signs of psychomotor regression, macrocephaly, ataxia, spasticity, hyperreflexia and involuntary movements [7].

This paper aims to describe two female patients with generalized chorea as the predominant clinical manifestation caused by leukodystrophy. The first case presents MLD with adult-onset chorea combined with cognitive-behavioral changes mimicking Huntington’s disease, while the second case is caused by L2HGA and the diagnosis had been established in the adulthood.

In addition, the description of involuntary movements in these disorders in the literature is presented as well.

## Methods

This case report includes two individuals. Participants were recruited based on clinical and radiological features combined with genetically proven pathogenic variants in the *ARSA* and *L2HGDH* genes, as determined by clinical exome sequencing (TruSight One panel). Their clinical and demographic information was collected while they were hospitalized at the Clinic for Neurology at the University Clinical Center of Serbia in Belgrade. Laboratory analysis, immunoserology, and brain MRI were performed for diagnostic purposes. Literature search was done through PubMed database (https://pubmed.ncbi.nlm.nih.gov/) from year of 1969 until July 21, 2024 using keywords: leukodystrophy AND chorea OR leukodystrophy AND movement disorder. We included articles that described case reports of patients (both adult and childhood-onseet) with leukodystrophy presenting with chorea or described as dyskinetic movements in patients with MLD or L2HGA, written in English language. Review articles, animal model studies or articles written in other languages were excluded.

## Results

### Case report 1

**Figure 1:**
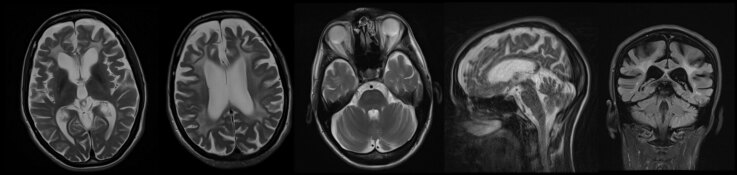
Bilateral symmetrical T2-weighted/FLAIR hyperintensities, together with global brain atrophy in patient carrying pathogenic variant in *ARSA* gene (Case 1).

Here we report a case of a young adult female who started developing behavioral changes at the age of 28 in the form of expressed jealousy, lying, and stealing things. Six years later her family members noticed involuntary movements of the head. She came to our clinic for the examination when she was 40 years of age and complained of weight loss, experienced instability while walking, and involuntary movement of her limbs, face, and trunk. Family history was negative for neurological disorders. The neurological examination showed generalized chorea with vocal tics, postural tremor of the hands, and voice. In addition to the above, she presented with dysarthria, wide gait, and mild dysmetria of the limbs (Video 1). The rest of the neurological examination was without impairment. Neuropsychological examination revealed dysexecutive syndrome. Laboratory analysis, immunoserology, tumor markers, and copper in urine were unremarkable. Autoimmune (antibodies against surface antigens and against intracellular antigens) and infectious causes (analysis for Borrelia burgdorferi, Human immunodeficiency virus and Treponema pallidum) were also excluded. As for the genetic testing, first, we excluded the presence of repeat expansions in *HTT, TBP, ATN1*, and *c9orf72* gene. MRI results showed evidence of bilateral symmetrical hypointensities in T1-weighted, while hyperintensities in T2-weighted/FLAIR, together with global brain atrophy (Figure 1). Considering these findings we performed the analysis of the arylsulfatase activity in leukocytes and the activity was slightly reduced to 3.2 nmol/h/mL (reference range is 3.6–9.4 nmol/h/mL).

**Video 1:** The clinical findings (generalized chorea, gaze impersistence, tongue protrusion, and ataxia) in patient carrying pathogenic variant in *ARSA* gene (Case 1).

The confirmation of the diagnosis was made with clinical exome sequencing which revealed two pathogenic variants c.542T > G (p. Ile181Ser) and c.763G > A (p. Glu255 Lys) in exon 3 and 4 of the *ARSA* gene (NM_001085425.3), respectively, which was later confirmed by Sanger sequencing.

### Case report 2

A 42-year-old female patient came to our outpatient clinic for examination with a history of progressive gait impairment, speech disturbance, and involuntary movements of the extremities. She was born as the first child from an uneventful pregnancy, delivered via spontaneous, uncomplicated vaginal delivery at term. Early psychomotor milestones were reached on time. At the age of two, the patient suffered febrile seizure and was prescribed Phenobarbital which she was taking for a year. No repeated seizures were observed after discontinuation of antiseizure medication. Her toddler and early preschool years were otherwise unremarkable.

At the age of five, her parents noticed a mild, irregular tremor of both hands which worsened when she started primary school. During the first grade, the teacher reported learning difficulties, lack of concentration, and the patient’s handwriting was impaired. She finished primary school and discontinued education afterward. Further, at the age of 22, she had her first bilateral motor seizure with consciousness impairment, and a year later, she suffered another seizure. Antiseizure therapy with sodium valproate was initiated, but she continued having repeated seizures until levetiracetam was introduced. Around the same time, involuntary movements of the face and hands were present with developing gait instability.

**Figure 2:**
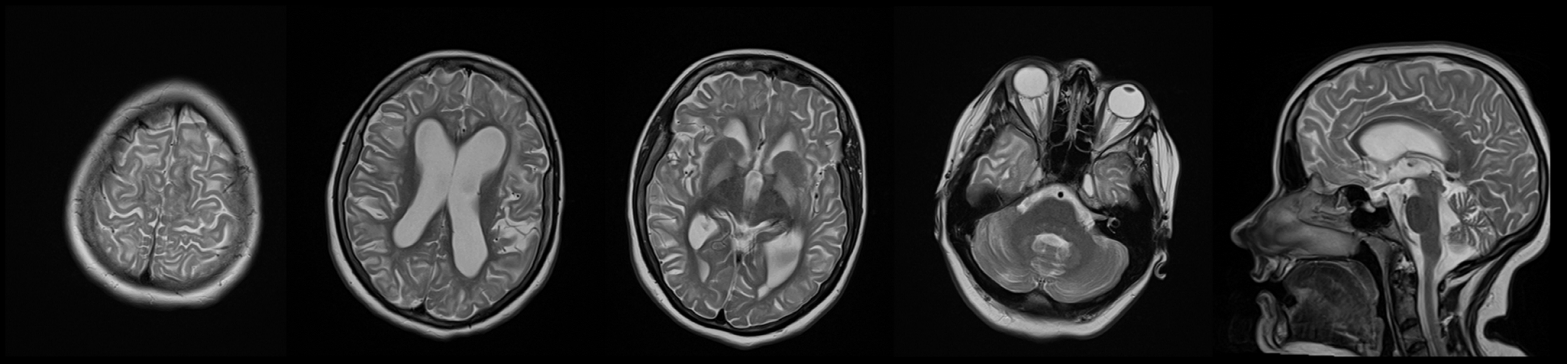
T2-weighted hyperintensity involving the subcortical white matter, globus pallidus, and dentate nucleus of the cerebellum in patient carrying pathogenic variant in *L2HGDH* gene (Case 2).

Her family history was positive for these disorders and her brother had the same symptoms and died at an early age. At the admission to our center, the neurological examination showed dysarthria, mild head tremor, facial grimacing, anterocollis, dystonia of the hands and feet with mild generalized chorea mainly affecting the trunk (Video 2). In addition, mild dysmetria of the limbs was noted bilaterally. Her gait was wide-based, with a dystonic posture of her left hand and left foot. Brain MRI showed T2-weighted image hyperintensity involving the subcortical white matter, globus pallidus, and dentate nucleus of the cerebellum; these abnormalities were considered highly suggestive of L2HGA (Figure 2). Metabolic screening of urine came back positive for l-2-hydroxy glutaric acid (value was 92 mmol/mol creatinine) using a gas chromatography/mass spectrometer, while normal values are up to 56 mmol/mol creatinine. Clinical exome sequencing revealed homozygous frameshift variant c.114_115del (p. cys38trpfsTer3) in the *L2HGDH* gene.

**Video 2:** The clinical findings (dysarthria, mild head tremor, facial grimacing, anterocollis, dystonia of the hands and feet with mild generalized chorea mainly affecting the trunk) in patient carrying pathogenic variants in *L2HGDH* gene (Case 2).

**Figure 3:**
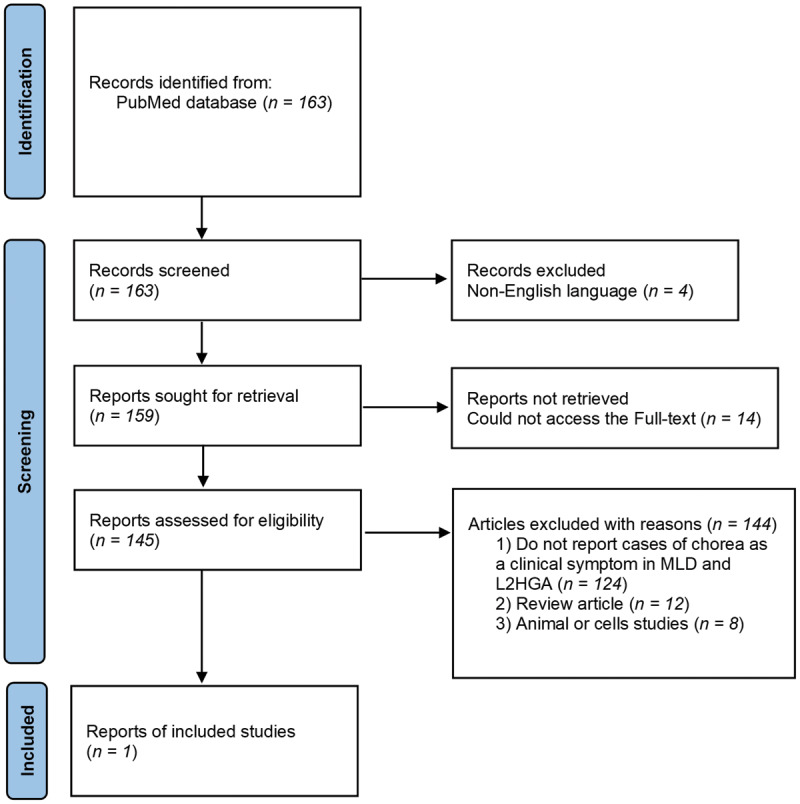
Article selection flow chart. L2HGA, L-2-hydroxiglutaric aciduria; MLD, Metachromatic Leukodystrophy.

A literature search was conducted on PubMed to identify case reports of leukodystrophy patients (with either adult- or childhood-onset) presenting with chorea or dyskinetic movements in MLD or L2HGA. The search resulted in 163 articles, but only one in the end described the phenotype suggestive of dyskinetic movement disorder as illustrated in Figure 3 [8,9].

## Discussion

In this paper, we have shown that MLD may be presented in adulthood as a Huntington-like phenotype (HD-like phenotype), which has not been described in literature so far. In addition, we have provided a description of choreodystonic movements in a patient with an earlier onset of L2HGA confirming that chorea might be a significant part of the clinical manifestations in other hereditary leukodystrophies as well. There have already been descriptions of certain leukodystrophies that may be manifested as an HD-like phenotype, such as the rare Gordon Holmes syndrome which is characterized by hypogonadotropic hypogonadism, ataxia, cognitive decline, chorea, and signs of cerebral atrophy on the MRI of the brain [1,10]. We showed that MLD has to be taken into consideration as differential diagnosis of HD-like phenotype and the hint could be MRI findings with symmetrical T2-weighted hyperintesities, which in adults starts in the rostral portion of the corpus callosum and frontal lobes [11].

Mutation in *ARSA* gene leads to decreased activity of arylsulfatase and consequently to demyelination and accumulation of cerebroside sulfate in the central and peripheral nervous system, and visceral organs [2,12]. There are three phenotypes of MLD depending of age at onset. Late infantile type has the worst prognosis with regression in motor skills, walking difficulties, seizures, ataxia, hypotonia and optic atrophy. Juvenile-onset shows a slightly slower progression of the disorder, whereas in adulthood the disorder most frequently manifests itself in behavioral changes, dementia, and psychosis [5]. The dominant difficulties are attention disorders, processing speed, executive dysfunctions, which is all typical of disorders of frontal-subcortical circuits, so the adult forms are frequently being wrongly diagnosed as psychoses or frontotemporal dementia [13,14]. Apart from the typical hyperintensity on MRI, predominantly in the frontal white matter, the changes in basal ganglia have also been described as a part of this disorder, which may explain the appearance of chorea and other involuntary movements in these patients. So far, one paper have stated that patients with MLD have the phenotype suggestive of dyskinetic movement disorder, while two more reported that patients might show chorea and dystonia, but without describing the actual cases [6,8,13].

Measuring the activity of arylsulfatase within leukocytes can indicate MLD and should be distinguished from pseudodeficiency where the values are 10–20% of the normal ones, whereas in MLD patients these values are far lower [15]. However, in our patient, this parameter was only slightly lower indicating the possibility that in atypical cases with a later onset, the values will not be reduced to such an extent. Residual activity of the enzyme is not the same in all cell types, and clinicians should bear in mind that it is practically impossible to measure a true residual activity, as leukocytes are not the main type of cells affected in MLD [16].

MLD is a genetically heterogeneous disorder, the most frequently described are missense variants and in a smaller number of cases splice-spite, deletions, or nonsense [17]. While the patients with an earlier onset have a higher phenotype uniformity, those with a later one show a greater diversity and earlier papers have suggested that these differences may arise from variable efect on the activity of the arylsulfatase enzyme [18,19]. Namely, in patients who have both null allele (0-allele), coding inactive arylsulfatase, a serious infantile form of the disorder is being developed. One allele, that provides a small amount of the residual activity of the arylsulfatase enzyme (R allele), leads to the juvenile, intermediary form of the disorder, whereas, following the principle of genetically dose-dependent effect, two R alleles when combined lead to a milder adult form of the disorder [17]. To the best of our knowledge and based on our literature review, this combination of variants in the ARSA gene has not been reported in the literature for our patient. According to functional analysis reported before, both variants individually lead to reduce activity of arylsulfatase enzyme, without completely eliminating activity [5,20,21]. As it was shown before in the literature, in parallel to the phenotype, certain variants lead to different enzyme activity, but the range was wide and it included overlapping which led to a conclusion that the MLD diagnosis cannot be made solely on the low level of the arylsulfatase activity, but it has to be confirmed through a genetic analysis or the sulfatide excretion in urine. If the enzyme’s activity is normal, and the clinical presentation and MRI indicate MLD, it is necessary to use genetic analysis to exclude saposin B deficiency which may lead to a similar phenotype [11].

Even though a significant number of centers nowadays have the possibillty of NGS, suspecting a certain disorder accelerates variants analysis by focusing the attention to a certain genes. Furthermore, the right clinical direction provides the possibility of single gene sequencing a useful way of making the genetic diagnosis.

Establishing the diagnosis of MLD is important as the new genetic therapies, such as atidarsagene autotemcel, based on the lentivirus to correct the altered gene in the stem cells which are firstly obtained from a patient and then returned, has beed approved by European Medicines Agency. A significant effect is reached if applied early in children when it comes to maintaining cognitive and motor functions and slowing down demyelination as well as cerebral atrophy [22]. So far, the medication has only been effective in asymptomatic late-infantile or early-juvenile forms, as well as early symptomatic early-juvenile forms.

Chorea has also been described in other forms of inborn errors of metabolism, such as organic acidurias [3], especially glutaric aciduria type 1, but it is rarely predominant signs and has not been clearly described in L2HGA so far. L2HGA is an AR disorder caused by pathogenic variants in *L2HGDH* gene encoding a Flavin Adenine Dinucleotide-(FAD)-dependent L-2-hydroxyglutarate dehydrogenase [23]. The disease usually begins in childhood, with macrocephaly, epilepsy, developmental delay, ataxia and involuntary movements [24]. Spasticity, hypotonia and behavioral changes were also mentioned. In the overview of 61 cases, Steenweg et al, described that 38% had movement disorders but without specifying which type they had. Besides limb dystonia, postural tremor and myoclonus, there is a description of writer’s cramp as initial manifestation of L2HGA [24]. We now emphasize that chorea associated with generalized dystonia can also be part of clinical manifestation. Malaquias et al recently expanded L2HGA phenotype and with description of two siblings with atypical parkinsonism and MRI basal ganglia iron deposition [7].

The diagnosis relay on brain MRI pattern as well as high levels of L2-hydroxyglutaric acid level in urine [24]. MRI findings predominantly show T2-weighted hyperintensities involving subcortical white matter sparing the periventricular regions. There are also hyperintense T2-weighted signal changes in the dentate nucleus, globus pallidus and caudate nucleus [25,26].

L-2-hydroxiglutaric acid is a disorder that is most frequently considered in childhood, but we have shown that this diagnosis needs to be taken into consideration in adulthood as well with a phenotype that predominantly manifests itself not only as dystonia but also as chorea. Although treatment option with riboflavin and levocarnitine have limited effect, recognizing this disoder is of great importance since they may have a role in therapy [27]. Beside that, establising diagnosis is important because patients frequently have associated brain tumors such as glioblastoma and neuroectodermal tumors [28].

The number of genes involved in the genetics of leukodystrophy and vascular leukoencephalopathies is continually growing and the medical literature shows the attempts to make algorithms, following the clinical manifestations and the specific patterns of hyperintensity on brain MRI, which may help us to follow the right clinical orientation [2]. Our cases indicate that involuntary movements such as generalized chorea should be included in the phenotype of the adult form and that MLD can give HD-like phenotype. On top of the above, chorea and dystonia are a part of the clinical manifestatons in other hereditary leukodystrophies as well, even those with a classic beginning in childhood.

Descriptions of these cases present the diversity in the clinical manifestations of leukodystrophies, together with chorea which may occur not only in classic infantile forms for which involuntary movements have already been described but also in adult forms of metachromatic leukodystrophy with HD-like phenotype which should be timely recognized.
